# Unusual anatomical variations of the hepatic arteries and bile ducts: What are the surgical implications

**DOI:** 10.4314/ahs.v22i3.74

**Published:** 2022-09

**Authors:** Nathalie Umugwaneza, Fidele Byiringiro, Paul Ndahimana, Andrew Ivang, Martin Nyundo, Faustin Ntirenganya, Julien Gashegu

**Affiliations:** 1 University of Rwanda, Department of Surgery; 2 University of Rwanda, Clinical Anatomy Unit

**Keywords:** Hepatic artery, Bile duct, variation, Surgical implication

## Abstract

**Introduction:**

The knowledge of anatomy is essential for surgical safety and impacts positively on patients' outcomes. Surgeons operating on the liver and bile ducts should keep in mind the normal anatomy and its variations as the latter are common.

**Case Presentation:**

We conducted a structured surgical dissection course of the supra-colic compartment of the abdominal cavity on 2nd and 3rd October 2020. While dissecting a 46years-old male cadaver, we encountered unusual anatomical variations of the hepatic arterial branching, the biliary tree, and arterial supply to the common bile duct. The common hepatic artery was dividing into two branches: a common short trunk for the left hepatic artery and the right gastric artery (hepato-gastric trunk) and a common trunk for the right hepatic artery and gastroduodenal artery (hepato-gastroduodenal trunk). The right hepatic duct was duplicated with a main right hepatic duct and an additional smaller duct. The bile duct was supplied by an artery coming from the abdominal aorta.

**Conclusion:**

We described three unusual anatomical variations: a variation of the hepatic arteries branching pattern, an aberrant right hepatic duct, and blood supply to the bile duct from the abdominal aorta. Surgeons should be aware of these rare variations.

## Introduction

Sound knowledge of normal anatomy and its variations is fundamental to hepatic and biliary surgery 1. The vascular supply to the liver and biliary tree comes from the celiac trunk (CT), the first major branch of the abdominal aorta 2. The CT originates at the level of the 12th thoracic vertebra and trifurcates into the left gastric, splenic, and common hepatic arteries to supply derivatives of the foregut 2. True trifurcation of the CT is the classical presentation found in nearly two-thirds of the population 3,4. However, variations to the branching pattern of the CT are common, including bifurcation, presence of additional branches as seen in the celiacomesenteric trunk, and complete absence of the CT, which is the rarest variation 3,5.

Hepatic arterial anatomy also has many variations, and these are highly relevant for radiological and surgical interventions. Michel's classification 6 and Hiatt's classification (a modification of Michel's description) 7 are the most common ways to define hepatic arterial branching patterns. Type I Michel's class is considered the normal pattern where the common hepatic artery arises from the CT, divides into the proper hepatic artery and gastroduodenal artery, and the proper hepatic artery terminates into right and left hepatic arteries 6. In total, Michel's classification has ten types representing different arterial variations as thoroughly described by the authors 6. Among described variations, Michel's type III variant (replaced right hepatic artery) is the most common, as seen in reports from different authors 6–9.

Additionally, the extrahepatic biliary tract frequently presents with varied anatomy, and the classical configuration of hepatic ducts is described in 60% of the population 10. Therefore, detailed knowledge of anatomical variations, including rare ones, is crucial to surgeons performing procedures on the liver and biliary tree in order to properly identify structures and prevent iatrogenic injuries. We aim to raise awareness of rare variations by reporting an unusual pattern of hepatic artery branching, bile ducts branching, and blood supply to the bile duct from the abdominal aorta.

## Case Presentation

We report a case of unusual hepatic artery and bile duct anatomical variations encountered during a cadaver anatomy dissection course. This course was delivered to surgery residents at the University of Rwanda, Clinical Anatomy Laboratory on October 2nd and 3rd, 2020 in Huye Campus, Rwanda. The surgical dissection course was designed by the Clinical Anatomy Unit in collaboration with the Department of Surgery. Trainers included consultant surgeons and anatomy experts. The dissection course received approval and accreditation from the Rwanda Medical and Dental Council.

Dissection was performed on a 46-year-old male deceased a year back from an unknown cause. The cadaver was embalmed through the femoral artery by injecting five liters of Dr Dankemeyer solution (a formula available at the laboratory) and conserved in the freezer up to its use. The dissection was about the supra-colic compartment of the abdominal cavity, which was accessed using three conventional incisions of the abdomen: a supra-umbilical median incision and two oblique incisions from the umbilicus to the anterior superior iliac spines. While dissecting the celiac trunk and its branches, we found the celiac giving, as commonly observed, the left gastric artery, the common hepatic artery, and the splenic artery.

**Figure 1:**
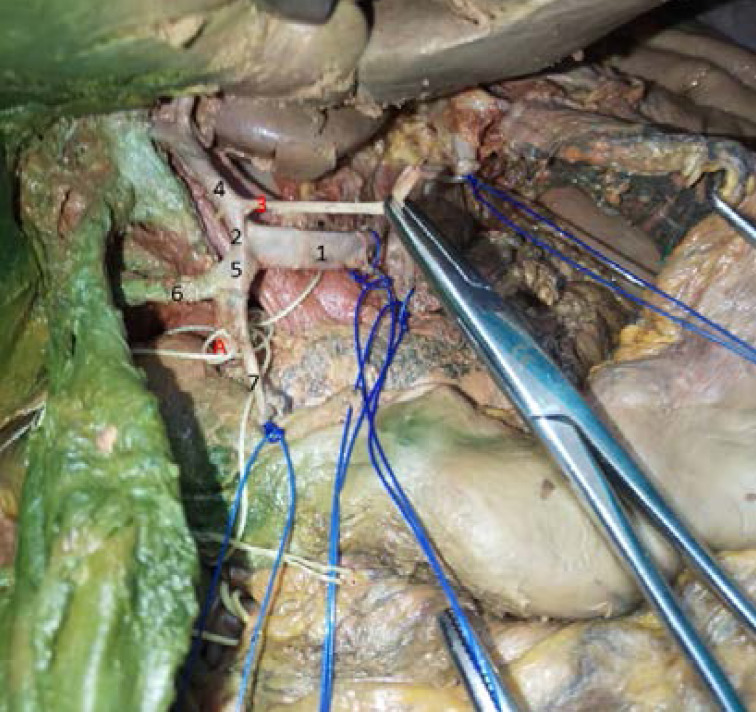
Hepatic arterial anatomy variation. 1. Common hepatic artery. 2. Hepato-gastric trunk. 3. Right gastric artery. 4. Left hepatic artery. 5. Hepato- gastroduodenal trunk. 6. Right hepatic artery. 7. Gastro-duodenal artery. A. Common bile duct.

**Figure 2:**
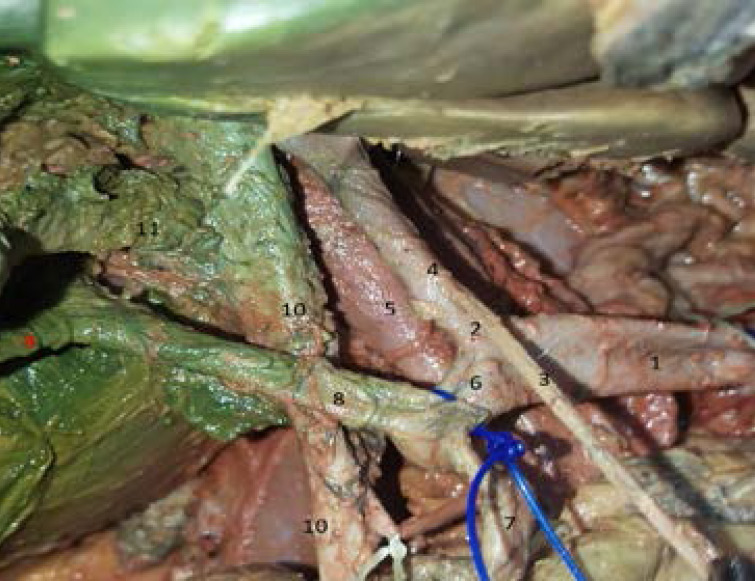
Common hepatic artery branches. 1. Common hepatic artery. 2. Hepato-gastric trunk. 3. Right gastric artery. 4. Left hepatic artery. 5. Portal vein. 6. Hepatogastroduodenal trunk. 7. Gastroduodenal artery. 8. Right hepatic artery. 9. Cystic artery. 10. Common bile duct. 11. Cystic duct.

Contrary to what is commonly seen, the common hepatic artery was dividing into two branches: a common short trunk for the left hepatic artery and the right gastric artery (hepatogastric trunk) and a common trunk for the right hepatic artery and gastroduodenal artery (hepato-gastroduodenal trunk) (Figure 1). The right hepatic artery was passing on the right posterior side of the cystic duct and giving the cystic artery (Figure 2).

**Figure 3:**
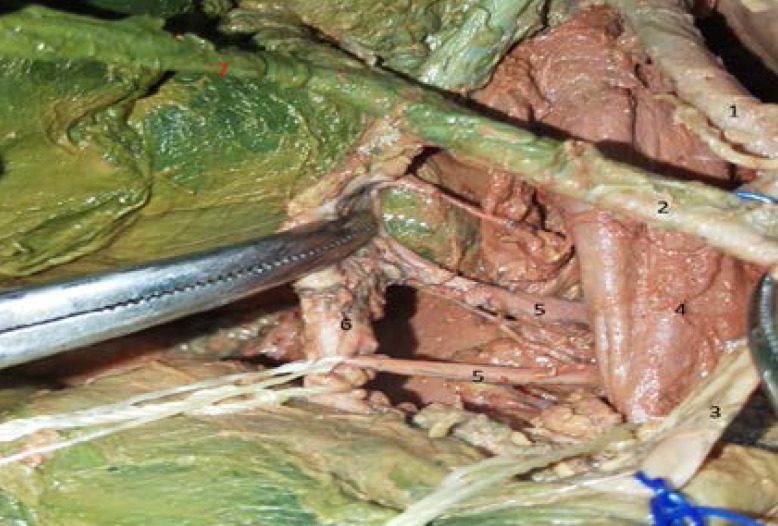
Variation of bile duct blood supply. 1. Left hepatic artery. 2. Right hepatic artery. 3. Gastro-duodenal artery. 4. Portal vein. 5. Artery for the bile duct, superior and inferior branches (branching from the aorta). 6. Common bile duct. 7. Cystic artery

The bile duct was supplied by an artery, the biliary artery, coming from the abdominal aorta passing between the inferior vena cava and the portal vein. This biliary artery was dividing into two branches, the superior biliary branch that supplied the proximal part of the bile duct and the inferior biliary branch that supplied the distal part of the bile duct (Figure 3).

**Figure 4:**
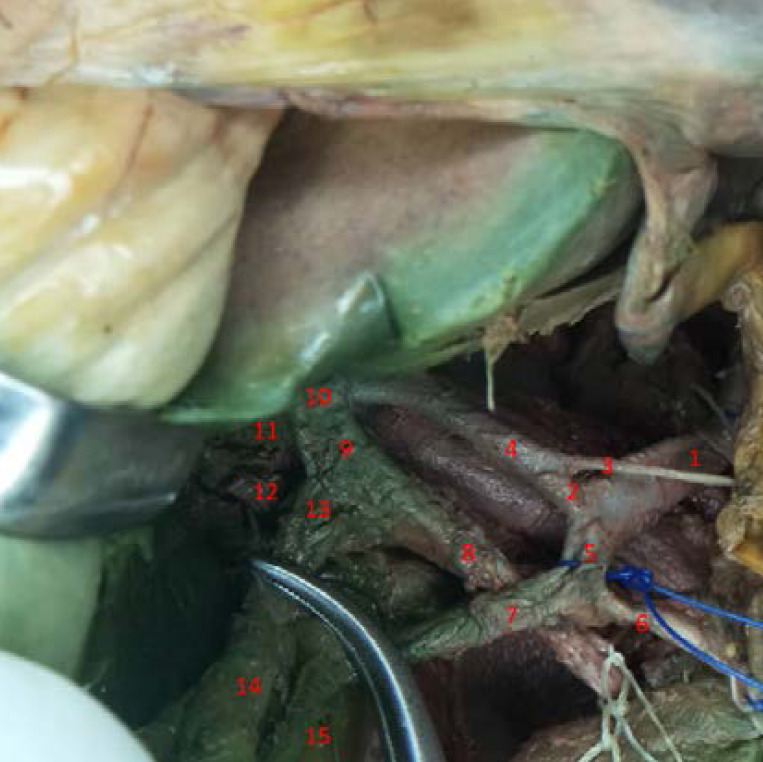
Variation of bile ducts. 1. Common hepatic artery. 2. Hepato-gastric trunk. 3. Right gastric artery. 4. Left hepatic artery. 5. Hepato-gastroduodenal trunk. 6. Gastroduodenal artery. 7. Right hepatic artery. 8. Common bile duct. 9. Common hepatic duct. 10. Left hepatic duct. 11. Right hepatic duct. 12. Additional right hepatic duct. 13. Hepatocystic duct. 14. Cystic duct. 15. Cystic artery.

In addition, the bile ducts branching presented an unusual pattern. The right hepatic duct was duplicated, a main right hepatic duct, and an additional much smaller duct. The main right hepatic duct was fusing with the left hepatic duct to form the common hepatic duct. The additional (aberrant) right hepatic duct fused with the cystic duct to form a short hepatocystic bile duct which joined the common hepatic duct to form the common bile duct (Figure 4). The calot triangle boundaries were the common hepatic duct on the left, the cystic duct on the right, and the inferior border of the liver. Most worrying was that the content of this triangle was not the usual cystic artery but the aberrant right hepatic duct (Figure 4).

## Discussion

Failure to recognize anatomic variations while performing procedures in the hepatobiliary region can result in major complications. Therefore, clinicians should master the relevant surgical anatomy before engaging in procedures involving the liver and biliary tree in order to minimize adverse outcomes. We are reporting three uncommon variations: variation of the hepatic arteries, variation of bile ducts branching pattern, and the blood supply to the common bile duct from the abdominal aorta. Radiologists and surgeons need to be acquainted with these rare variations.

In the present case, we observed a common hepatic artery that divided into two trunks: the hepato-gastric trunk (for the left hepatic artery and the right gastric artery), and the hepato-gastroduodenal trunk (for the gastroduodenal artery and right hepatic artery). The proper hepatic artery was absent. As the right and left hepatic arteries are branches of the common hepatic artery, which in turn rises from the CT, our case can be classified in the Michel's and Hiatt's type I categories 7. However, the branching pattern is different from what is classically described in textbooks (that the right and left hepatic arteries branch from the proper hepatic artery) 1. The literature shows that hepatic arterial variations are common, as evidenced by multiple reports that have described anatomical variations in 20.9%–45% of cases 6,8,11,12. Variations previously described in which the proper hepatic artery was absent include: common hepatic artery trifurcation into gastroduodenal artery, left hepatic artery, and right hepatic artery 11,13, and the common hepatic artery dividing into right and left hepatic arteries, with the gastroduodenal artery originating from the right hepatic artery 14. Moreover, Shetty et al. 15 have reported a rare case in which the common hepatic artery gave rise to an additional right hepatic artery coursing in the Calot's triangle, putting it at an increased risk for iatrogenic injuries.

Another variation we found in the current case is an aberrant right hepatic duct which joined the cystic duct to form a short duct that drained into the common hepatic duct.

As a result, the content of the Calot's triangle included this aberrant right hepatic duct. Previous reports show that anomalous ducts are quite common, occurring in 12% of cases on average 16. Furthermore, anomalous ducts are found within Calot's triangle in approximately 85% of cases, making them vulnerable during procedures such as cholecystectomy 1. The literature also shows that most aberrant ducts rise from the right lobe of the liver^1^. Moreover, aberrant right hepatic ducts commonly join the cystic duct or the common hepatic duct 1. Rarely, these aberrant ducts unite with the gallbladder or common bile duct 1. Inversion of Calot's triangle is an extremely rare variation reported by Nayak 17, whereby the cystic duct joins the left side of the common hepatic duct.

The third variation we observed was blood supply to the common bile duct from an artery originating directly from the abdominal aorta. Usually, blood supply to the bile duct comes from branches of the gastroduodenal artery, right hepatic artery, and cystic artery 18. To our knowledge, there are no previous reports of an artery to the bile duct rising directly from the aorta. Surgeons should be aware of this rare source of blood supply to the bile duct and possible unexpected bleeding upon opening the duct. Furthermore, detailed knowledge of the biliary tree blood supply is crucial during liver transplantation to decrease the risk of ischemic complications 19.

Our findings carry numerous surgical implications. Firstly, surgeons performing procedures on the liver, biliary tree, and upper gastrointestinal tract need to look out for anatomic variations as they are common in this region. Meticulous dissection and proper identification of structures before any clamping and division are always warranted. Furthermore, pre-operative imaging studies such as computed tomography (CT) angiography and magnetic resonance (MR) cholangiopancreatography should be done as indicated to guide operative planning 20,21. For instance, finding anomalous branching patterns, replaced or accessory hepatic arteries requires extra caution to prevent injury and ischemia during surgeries on the biliary tree, liver resections, and liver transplantation 21.

Lastly, Calot's triangle is a very important landmark during both open and laparoscopic cholecystectomy. The presence of variant anatomy increases the risk of biliary and vascular injuries, more so during laparoscopic procedures^21^,^22^. Therefore, surgeons performing cholecystectomy should be aware of the possible variations in the contents of Calot's triangle, such as an aberrant right hepatic duct. Sound knowledge of clinical anatomy, the anticipation of possible anatomic variations, and careful inspection of Calot's triangle contents before ligation can decrease the risk of iatrogenic injuries.

## Conclusion

We described three unusual anatomical variations: a variation in the branching pattern of hepatic arteries, a variation in the branching pattern of bile ducts, and the blood supply of the bile duct from the abdominal aorta. The knowledge of hepatic arterial and biliary anatomy variations is critical to ensure surgical safety. The presented case invites surgeons to properly do pre-operative assessment and careful dissection intra-operatively to prevent inadvertent injury to structures that can lead to major complications.
